# The first reader: Montaigne and the body’s particularity

**DOI:** 10.1017/S1478951526102995

**Published:** 2026-06-23

**Authors:** Henry Bair

**Affiliations:** Wills Eye Hospital, Philadelphia, PA, USA

In 1578, at the age of 45, Michel de Montaigne began to pass kidney stones. The pain was extraordinary, the same pain his father had endured before him. Montaigne writes about this inheritance in “Of the Resemblance of Children to Their Fathers,” and his astonishment is at once medical and metaphysical. His father had been healthy through youth and middle age. The stone came on him only late and killed him within a few years. Then, somehow, it crossed the gulf of generation and returned in the son decades later. What does it mean to carry a father’s pain inside you, dormant, before it is yours? Montaigne does not answer with theology or with humoral theory. He answers, gradually and over more than a decade, with a method of attention: the *essai*, the trying.

That the patient is an authoritative knower of her own condition has been argued for years, by phenomenologists of illness, by narrative medicine programs, by the literature on epistemic injustice in healthcare. What Montaigne offers is something the argument has not made easy to practice. He attended to his body without diagnostic ambition. He was not trying to figure out what was wrong; he was trying to know it. Clinical attention, the kind physicians are trained for, is structurally diagnostic. It scans for patterns, narrows toward category, and organizes details around hypothesis. This is what makes it useful. It is also what makes Montaigne’s procedure almost impossible for a clinician to perform without feeling that she has stopped working. That feeling, I want to suggest, is the discipline. It is what we have not learned to tolerate.

Montaigne is not the first to write about his body. Galen left case histories. Augustine wrote a *Confessions* that included his physical infirmities. His older contemporary Girolamo Cardano drafted *De Vita Propria* in 1575, an autobiography of unsparing self-examination structured in the Suetonian manner, by character and theme rather than by sustained somatic attention; it would not be published until 1643. What is distinctive in Montaigne, and what emerges only after the stones came, is the method of suspended diagnosis. The first book of *Essais* (1580) contains the expected materials of late Renaissance humanism: Stoic consolations, military exempla, Plutarchan commonplaces. The somatic method belongs to the third book of 1588 and to the post-1578 additions to the first 2. It tracks the disease.

You can see the method at work in the essay “Of Experience” if you watch what Montaigne refuses to do. He records that physicians contradict one another on every question of regimen, that the same abstinence one credits with saving a patient another blames for killing him. One reasonable conclusion would be to demand a better system. Montaigne draws the opposite conclusion: that no system, however orderly, can stand in for a particular body. He attends to his sleep, his digestion, his appetite, the rhythm of his stones, with the patience of a man who has decided that knowing them is its own end. At one point he asks, half-seriously, to be woken from sleep “to the end that sleep itself should not escape me thus stupidly.” There is a kind of intellectual courage in this, in the refusal to convert observation into theory the moment theory is available.

The famous interrogative *que sais-je?* (“what do I know?”) inscribed on the wooden beams of his library among passages from Sextus Empiricus and other Pyrrhonian sources, is not false modesty. It is an epistemic discipline, the skeptic’s question turned inward. The genealogy matters because it complicates the standard picture of Montaigne the reasonable anti-physician. He did hate doctors, and his complaints about 16th-century medicine were well-founded; it was a hodgepodge of incompatible theories — Galenic, Paracelsian, astrological, folk — and a physician of that century could examine a patient and reach almost any conclusion he liked. But Montaigne’s posture was not simply empirical; it was partly an inheritance from Sextus, a skepticism toward systematic doctrine, applied with particular force to the doctrine that claimed jurisdiction over his body. He would not have trusted a more disciplined medicine either, or at least not without the same suspicion. What he would have wanted from such a medicine, and what he showed by example, was an attention that observed before it classified.

Four centuries on, that requirement is the one we are still struggling with. Medicine has spent the last 2 decades trying to recover something like Montaigne’s position: precision medicine, n-of-1 trial designs, patient-reported outcome measures, shared decision-making, all real efforts to put the particular body back at the center of the encounter, all of which have produced real gains. But the diagnostic instinct is the gravity that pulls every medicine, including a good one, away from the body in front of it. We listen, and even as we listen, we are sorting. The intake form renders the patient into a checklist before the clinician sees her. The visit produces a coded diagnosis before it produces a description. Montaigne’s caution is not against bad medicine. It is against a gravitational pull that good medicine has not yet learned how to suspend, even briefly, even when suspending it is what the patient most needs.

A patient I saw recently has bilateral geographic atrophy from age-related macular degeneration. The damage was irreversible, and area of atrophy had grown at roughly the rate the literature would have predicted, and by that metric she was stable. I had the reassuring sentence already forming when she told me, almost in passing, that she had stopped recognizing her husband’s face across the kitchen and that she had begun setting the table by touch because she could no longer find the edge of her plate. I held the sentence I had been able to say. The area metric had told me how much retina had been lost; it had not told me where. The atrophy had reached the foveal center, and the millimeters that mattered to her life were the millimeters my summary had treated as equivalent to any others. Her description had not replaced the data; it had reorganized my reading of it. Her attention to her own vision had been better than mine, and earlier, and freely given, and I had nearly missed it because the number had been a number.

There is a moment in “Of Practice,” when Montaigne is describing his return to consciousness after a fall from a horse, in which he recalls a strange sweetness at the threshold, almost a pleasure in the dissolution. The passage is often read as a report on dying when it is a report on syncope. What it does show, and what I think Montaigne understood with unusual clarity, is that the inside of bodily extremity does not reliably resemble its outside. He gave us the question 4 centuries before researchers on palliative medicine arrived with the data to answer it.

Montaigne died in 1592, of a peritonsillar abscess, 14 years after the stones first came. He had filled 3 books with the work of a life examined from inside. The achievement is humbling. We ask our patients to rate their pain on a scale of 10; he gave us a 1000 pages on the texture of his. We ask them to check the box for family history; he wrote an essay about what it means to inherit a disease across the silence of a generation.

I am not proposing that the *Essais* be included on a medical school reading list, though that wouldn’t be the worst outcome. I am arguing for a discipline the clinic does not by itself produce. The clinic is built to translate bodies into categories, and that translation is necessary. But the translation can begin too soon, and once it begins it tends not to stop. The minimum we owe a patient is the suspension, however brief, of the instinct that converts her into the case she resembles. We owe her, before the diagnosis, a reading. We are, more often than we realize, a second opinion on a manuscript she has been studying her whole life.

